# Visual outcomes and prognostic factors following 23G vitrectomy for vitreous hemorrhage in eyes with proliferative diabetic retinopathy

**DOI:** 10.5935/0004-2749.20230037

**Published:** 2022-03-08

**Authors:** Ioana Damian, Simona Delia Nicoară

**Affiliations:** 1 Department of Ophthalmology “Iuliu Haţieganu” University of Medicine and Pharmacy, Cluj-Napoca, Romania.; 2 Medical Doctoral School, University of Oradea, Oradea, Romania.; 3 Clinic of Ophthalmology, Emergency County Hospital, Cluj-Napoca, Romania.

**Keywords:** Diabetic retinopathy, Vitreous hemorrhage, Vitrectomy, Intravitreal injection, Visual acuity, Retinopatia diabética, Hemorragia vítrea, Vitrectommia, Injeção intravítrea, Acuidade visual

## Abstract

**Purpose:**

We aimed to evaluate the factors influencing the visual gain following pars plana vitrectomy for vitreous hemorrhage in patients with proliferative diabetic retinopathy.

**Methods:**

A retrospective study was conducted on 172 eyes of 143 consecutive patients with diabetes mellitus between January 2012 and January 2018. Demographic data, ophthalmological findings, surgery details, and visual outcomes were gathered after consulting the patients’ records. The main outcome measured was the improvement of best corrected visual acuity and the secondary outcomes measured were rebleeding and complications.

**Results:**

Best corrected visual acuity improved in 103 eyes (59.88%), worsened in 45 eyes (26.16%), and remained unchanged in 24 eyes (13.95%). Type 2 diabetes mellitus was significantly associated with better final best corrected visual acuity (p=0.0244). Previous treatment by pan-retinal laser photocoagulation or intravitreal bevacizumab determined better final best corrected visual acuity, but not significantly (p>0.05). Preoperative rubeosis iridis and neovascular glaucoma did not influence the outcomes. The lack of fibrovascular proliferation requiring dissection was a significant factor for better final best corrected visual acuity (p=0.0006). Rebleeding occurred in 37.1% of the eyes and it was not influenced by the antiplatelet drugs (p>0.05). Postoperative neovascular glaucoma was a negative prognostic factor (p=0.0037).

**Conclusion:**

The final best corrected visual acuity was influenced positively by type 2 diabetes mellitus and the absence of preoperative extensive fibrovascular proliferation and negatively by postoperative neovascular glaucoma.

## Abstract (pt)

**Objetivo:**

Avaliar os fatores que influenciam o ganho visual após vitrectomia via *pars plana* para hemorragia vítrea em pacientes com retinopatia diabética proliferativa.

**Métodos:**

Foi realizado um estudo retrospectivo de 172 olhos de 143 pacientes consecutivos com *diabetes mellitus* entre janeiro de 2012 e janeiro de 2018. Dados demográficos, achados oftalmológicos, detalhes da cirurgia e resultados visuais foram coletados através de consulta aos prontuários dos pacientes. A principal medida de desfecho foi o aumento da melhor acuidade visual corrigida e as medidas de desfecho secundário foram a recidiva da hemorragia e a ocorrência de complicações.

**Resultados:**

A melhor acuidade visual corrigida aumentou em 103 olhos (59,88%), diminuiu em 45 olhos (26,16%) e permaneceu inalterada em 24 olhos (13,95%). O *diabetes mellitus* tipo 2 foi significativamente associado a maiores valores finais da melhor acuidade visual corrigida (p=0,0244). O tratamento prévio por fotocoagulação panretiniana com laser ou bevacizumabe intravítreo determinou maiores valores da melhor acuidade visual final corrigida, mas não significativamente (p>0,05). A presença de rubeose iridiana pré-operatória ou de glaucoma neovascular não influenciou os desfechos. A ausência de proliferação fibrovascular com necessidade de dissecção foi um fator significativo para maiores valores da melhor acuidade visual final corrigida (p=0,0006). Ocorreu recidiva da hemorragia em 37,1% dos olhos e não foi influenciada por fármacos antiplaquetários (p>0,05). O glaucoma neovascular pós-operatório foi um fator prognóstico negativo (p=0,0037).

**Conclusão:**

O resultado final da melhor acuidade visual corrigida foi influenciado positivamente pelo *diabetes mellitus* tipo 2 e pela ausência de proliferação fibrovascular extensa no pré-operatório, e negativamente pela ocorrência de glaucoma neovascular pós-operatório.

## INTRODUCTION

Diabetic retinopathy (DR) is the leading cause of new-onset blindness in middle-income countries^(1)^. DR develops in nearly all patients with type 1 diabetes mellitus (T1DM) and in more than 77% of patients with more than 20 years of history of type 2 diabetes mellitus (T2DM). The prevalence of vision-threatening DR is 10.2% (28 million people)^(1)^. Its increasing prevalence determined the World Health Organization to include DR on the list of high-priority eye diseases and implement programs to prevent related blindness and visual impairment related^(2)^.

Non-clearing vitreous hemorrhage (VH) is a high-risk feature of proliferative diabetic retinopathy (PDR). Fortunately, there are approaches to address PDR and its complications, including pars plana vitrectomy (PPV), pan-retinal photocoagulation (PRP), and intravitreal anti-vascular endothelial growth factor (VEGF) injections. PPV is the most important component of treatment in VH, acting synergistically with other methods. Historically, the first indication for PPV was severe non-clearing VH in a diabetic patient^(3)^. In parallel with the progress of PPV technology, new indications for PPV in patients with DM emerged: tractional retinal detachment (TRD) involving or threatening the macula, combined tractional and rhegmatogenous retinal detachment (RRD), dense premacular hemorrhage, ghost cell glaucoma, macular edema with premacular hyaloid traction, and severe PDR^(4)^.

VH occurring in patients with PDR is frequently a complex scenario to the vitreoretinal surgeons. The visual outcome depends not only on the surgery itself but also on the severity and monitoring of the underlying systemic and local conditions. Starting from the complexity of these cases, our first specific objective was to analyze the factors potentially influencing the visual gain following 23G PPV, such as associated conditions, previous ocular treatment, additional surgical procedures to PPV (intravitreal injections and laser photocoagulation), and postoperative complications. Secondly, we aimed to evaluate the risk of rebleeding and its potential trigger factors.

## METHODS

This retrospective study was approved by the Ethics Committee belonging to “Iuliu Hatieganu” University of Medicine and Pharmacy, Cluj-Napoca, Romania. The protocol has been performed according to the Declaration of Helsinki. A total of 172 eyes belonging to 143 consecutive patients operated for VH due to DM over January 2012 and January 2018 were included in the study. All surgeries were performed by the same surgeon in the Department of Ophthalmology, Emergency County Hospital from Cluj-Napoca, Romania.

All information, such as demographic data, ophthalmological findings, surgery details, and visual outcomes, was gathered after consulting the patients’ records from pre-, intra-, and postoperative evaluations. The following inclusion criteria were used: non-clearing VH for over a month in a patient with DM, PDR at least in one eye, and follow-up for more than 3 months. The exclusion criteria were a history of PPV in the same eye, other macular pathology, ocular trauma, and advanced glaucoma. Preoperative assessment included best corrected visual acuity (BCVA), slit-lamp examination with +90 D lens, indirect ophthalmoscopy, and applanation tonometry. The main outcome measured was the improvement of BCVA following PPV and the secondary outcomes measured were rebleeding and other complications.

The final visual outcome was classified according to the evolution of BCVA as follows: improved, worse, or unchanged as compared to the baseline.

### Therapeutic protocol

#### Intravitreal injection

Intravitreal bevacizumab (IVB) was performed either before or during PPV. Preoperative IVB was administered according to the following protocol: after sterile preparation and draping, a volume of 0.05 ml (1.25 mg) of bevacizumab (Avastin, Genentech) was injected intravitreally, 3.5 mm away from the limbus, under topical anesthesia.

#### Pars plana vitrectomy

A single surgeon performed a 3-port 23-gauge trans­conjunctival sutureless vitrectomy using the Alcon Accurus surgical or the Alcon Constellation system. The infusion line was inserted in the inferotemporal quadrant. Using the vitreous cutter and a light pipe, a core vitrectomy was performed, followed by the induction of posterior vitreous detachment, unless it was not already present. If hemorrhage occurred intraoperatively, hemostasis was obtained by elevating the infusion pressure. According to the situation, aspiration of the VH, peeling, or en bloc dissection of the tractional membranes was carried out. Dissection was performed either with a 25 G forceps or with the vitreous cutter, and the bimanual technique was not applied. If needed, the retinal endolaser was completed. Phacoemulsification with intraocular lens insertion and PPV were combined when the lens opacity prevented the visualization of the posterior segment. At the end of the surgery, the vitreous cavity was left under Balance Salt Solution (BSS), air, or silicone oil 1000 cSt. Postoperative hemorrhage or rebleeding following the primary PPV was considered early- (<4 weeks after PPV) or late-onset (>4 weeks after PPV).

#### Statistical analysis

For the statistical analysis, p-value was calculated with Chi-square and Fisher exact test using Social Science Statistics (www.socscistatistics.com/tests) to identify the variables that were associated with the best outcomes. Numerical variables were summarized with means and percentages. P<0.05 was considered statistically significant.

## RESULTS

**Figure 1:**
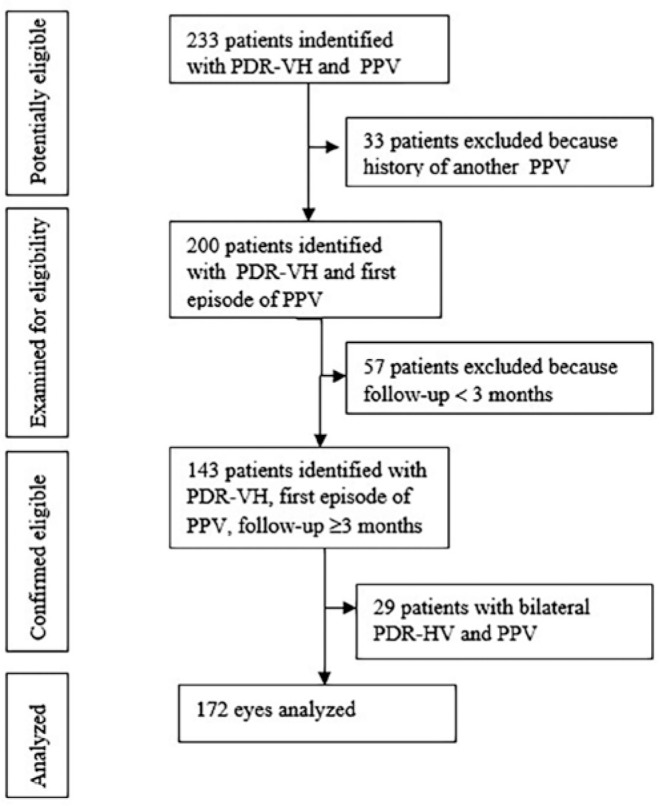
Patients’ selection process.

The flowchart of the patients’ selection process is illustrated in figure 1.

A total of 172 eyes belonging to 143 patients who underwent PPV for VH were included in this study. The average age was 60 years (min22 - max80). According to the gender distribution, there were 97 males (56.3%) and 75 females (43.7%) in our study group. T1DM was diagnosed in 21 patients (12.3%) with an average duration of 15.91 years (min 14 months - max 46 months). T2DM was diagnosed in 151 patients (87.7%) with an average duration of 15.55 years (min 2 months - max 48 years).

### Main outcome: BCVA

Initial preoperative and final postoperative BCVAs are illustrated in table 1.

**Table 1:** Initial preoperative BCVA and final postoperative BCVA

	NLP n (%)	LP n (%)	HM n (%)	CF n (%)	0.01-0.04 n (%)	0.05-0.09 n (%)	>0.1 n (%)
Initial BCVA	1 (0.58)	3 (1.74)	42 (24.4)	57 (33.1)	40 (23.2)	14 (8.13)	14 (8.13)
Final BCVA	8 (4.65)	4 (2.32)	23 (13.3)	19 (11.0)	38 (22.0)	27 (15.6)	45 (26.1)

BCVA= best corrected visual acuity, NLP= no light perception; LP= light perception; HM= hand motion; CF= counting fingers; n= number.

Preoperative BCVA was counting fingers in 57 patients (33.10%). Final postoperative BCVA was ≥0.1 in 45 patients (26.10%). The final visual outcome was classified according to the evolution of BCVA as follows: improved, worse, or unchanged, as compared to the baseline. BCVA improved in 103 eyes (59.88%), worsened in 45 eyes (26.16%), and remained unchanged in 24 eyes (13.95%). The evolution of BCVA is illustrated in table 2.

**Table 2:** The evolution of VA

Initial VA	Improved final VA n (%)	Worse final VA n (%)	Unchanged final VA n (%)
NLP	1 (100)	0	0
L.P.	1 (33.3)	0	2 (66.6)
H.M.	24 (57.1)	9 (21.4)	9 (21.4)
CF	46 (80.7)	7 (12.2)	4 (7.01)
0.01-0.04	25 (62.5)	11(22)	4(8)
0.05-0.09	2 (13.3)	9 (60)	4 (26.6)
>0.1	4 (28.5)	9 (64.2)	1 (7.14)

VA= visual acuity; LP= light perception; HM= hand motion; CF= counting fingers.

The gender did not influence the final BCVA (p=0.4154). The patients with T2DM had a significantly better final BCVA compared to the patients with T1DM (p=0.0244). No statistically significant correlation was found between the presence of comorbidities (high blood pressure, history of stroke, heart disease, dyslipidemia, neuropathy, or nephropathy) and the final BCVA (p>0.05). The treatment with antiplatelet drugs did not influence the final BCVA (p=0.8350). Regarding the hypoglycemic therapy, no difference emerged between the 2 groups (Table 3).

**Table 3:** Comorbidities and BCVA

Characteristics	Improved BCVA	Worse BCVA	Unchanged BCVA	p value
**Gender:** M/F	56/47	29/16	12/12	0.4154
**Type 1 DM/Type 2DM**	9/94	11/34	2/22	*0.0244*
**HBP:** yes/no	84/19	33/12	18/6	0.4832
**Stroke:** yes/no	7/96	5/40	1/23	0.5236
**Heart disease:** yes/no	37/66	12/33	4/20	0.1439
**Dyslipidemia**: yes/no	31/72	15/30	4/20	0.3262
**Neuropathy:** yes/no	19/84	5/40	4/20	0.5380
**Nephropathy:** yes/no	11/92	8/37	2/32	0.2397
**Antiplatelet drugs:** yes/no	32/71	13/32	6/18	0.8350
**Insulin:** yes/no	90/13	35/10	17/7	0.0969
**Oral antidiabetic drugs**	29/29	39/18	11/11	0.0989

M= male; F= female; DM= Diabetes mellitus; HBP= High blood pressure; BCVA= best corrected visual acuity.

The italicized p values indicate a statistically significant difference between groups.

Of the 172 eyes, 148 eyes (86%) had one episode of VH, 20 eyes (11,6%) had 2 episodes of VH, 2 eyes (1.16%) had 3 episodes of VH, 1 eye (0.58%) had 4 episodes of VH, and 1 eye (0.58%) had 6 episodes of VH. PPV for the recurrence of VH was necessary in 33.80% of cases. Multiple surgeries had no significant impact on the final BCVA (p>0.05).

In terms of the lens condition, pseudophakic eyes had better final BCVA compared to the phakic ones, but not statistically significant (p=0.1162).

PDR was treated before the occurrence of VH in 86 eyes (50%): in 50 eyes (29.06%) by PRP and in 36 eyes (20.93%) by IVB. These previously treated eyes had better final BCVA, but the difference was not statistically significant. However, 88% of the eyes that received laser for PDR before PPV needed endolaser during surgery. Eyes that did not require membrane dissection had significantly better final BCVA as opposed to the eyes in which the dissection was required (p=0.0006). Iatrogenic retinal breaks occurred in 2 eyes (1.16%). The eyes were left under silicone oil in 50%, air in 8.13%, and BSS in 41.8% of cases. The type of tamponade did not influence the final BCVA (Table 4).

**Table 4:** Preoperative and intraoperative characteristics related to the evolution of BCVA

Characteristics	Improved BCVA	Worse BCVA	Unchanged BCVA	p value
Phakia/Pseudophakia n/n	90/13	39/6	17/7	0.1162
Rubeosis: yes/no	4/99	2/43	3/21	0.2241
NVG: yes/no	4/99	0/45	0/24	
Treatment of PDR before PPV				
PRP: yes/no	29/74	15/30	6/18	0.7293
IVB: yes/no	25/78	7/38	4/20	0.4181
IVB preoperatively: yes/no	63/40	21/24	11/13	0.1604
PPV associated procedures				
Membrane dissection: yes/no	20/83	24/21	9/15	0.0006
Endolaser: yes/no	96/7	41/4	21/3	0.6399
Phacoemulsification: yes/no	7/96	1/44	3/21	0.2428
IVB intraoperative: yes/no	18/85	10/35	2/22	0.3504
Silicone oil	47	26	13	0.7222
Air	9	3	2	
BSS	47	16	9	

NVG= neovascular glaucoma; PPV= pars plana vitrectomy; IVB= Intravitreal Bevacizumab; PRP= pan-retinal photocoagulation; BCVA= best corrected visual acuity; BSS= Balance Salt Solution. The italicized p values indicate a statistically significant difference between groups.

### Secondary outcomes: postoperative complications and rate of rebleeding

Postoperative complications consisted of cataract (38 eyes, 22.09%), neovascular glaucoma (NVG; 15 eyes, 8.72%), endophthalmitis (1 eye, 0.58%), and RRD (1 eye, 0.58%). The incidence of cataracts following PPV varied with tamponade agents: 20.53% for silicone oil, 15.39% for BSS, and 3.8% for air, but without statistical significance (p>0.05).

Each complication was approached accordingly by surgical and/or medical treatment. None of the complications had a significant negative impact on the final BCVA. The absence of postoperative NVG had a significant positive impact on the final BCVA (p=0.0373). Phacoemulsification combined with PPV was not associated with a higher rate of postoperative NVG in our case series, where 2 of the 11 eyes with combined surgery developed NVG postoperatively (Table 5).

**Table 5:** Post PPV complications

Characteristics	Improved BCVA	Worse BCVA	Stable BCVA	p value
**Early rebleeding:** yes/no	14/89	8/37	2/22	0.5513
**Late rebleeding:** yes/no	20/83	15/30	5/19	0.1746
**NVG:** yes/no	5/98	8/37	2/22	*0.0373*

NVG= neovascular glaucoma; BCVA= best corrected visual acuity; PPV= pars plana vitrectomy.

The italicized p values indicate a statistically significant difference between groups.

Rebleeding occurred early (<4 weeks after PPV) in 13.9% and late (>4 weeks after PPV) in 23.2% of the eyes without significant influence on the final BCVA.

IVB was administered before PPV (average of 3.8 days) in 147 of the 172 eyes (85.46%) and at the end of PPV in the remaining 25 eyes (14.54%). A positive effect was proven in eyes that received IVB at 3.8 days in average before surgery in preventing early or late postoperative rebleeding and at the end of the surgery, without statistical significance between the two settings of IVB injection (p>0.05).

The administration of antiplatelet drugs (cyclooxygenase 1 (COX-1) inhibitors group, such as aspirin) did not influence the onset of early or late rebleeding (p>0.05; Table 6). Lower extremity amputation and the inability to control blood pressure were not associated with an increased risk of rebleeding.

**Table 6:** Risk of rebleeding

	Risk of early rebleeding			Risk of late rebleeding		
	Yes	No	p value	Yes	No	p value
**Antiplatelet drugs:** yes/no	5/19	46/102	0.3078	10/30	41/91	0.4622
**IVB before PPV:** yes/no	16/8	79/69	0.2245	18/22	77/55	0.1373
**IVB intraoperative:** yes/no	3/21	27/121	0.4915	11/29	19/113	0.5567
**Lower extremity amputation:** yes/no	2/22	26/122	0.2556	7/33	21/111	0.8112
**HBP:** yes/no	18/6	117/31	0.6538	35/5	100/32	0.1133

IVB= intravitreal Bevacizumab; PPV= pars plana vitrectomy; HBP= high blood pressure.

## DISCUSSION

The goal of PPV in VH caused by DM exceeds the clearing of the vitreous body, being directed mostly toward treating the underlying PDR. This study adds to the numerous publications in the field by presenting our original algorithm to treat VH in patients with DM. The analysis of the outcomes and prognostic factors from our research outlines an original strategy to manage VH in patients with DM, which could set the stage for further research.

### Type of diabetes

Our data confirm that patients with T1DM have a worse disease course, which may be explained by the poorer metabolic control. Other reports found that the incidence of sight-threatening DR was higher in T1DM^(5)^. A prospective study carried on 3980 patients with T1DM found a cumulative incidence rate of diabetic vitrectomy of 2.9% after 10 years of follow-up, especially for those with HbA1C >75 mmol/mol^(6)^.

### BCVA at baseline

The eyes with the lowest BCVA before PPV (hand movement, counting fingers, and 1/50 vision) benefited the most from the surgery in our study. This finding is in agreement with the results of another study demonstrating that the progress of visual acuity has been greater if the presenting vision was worse^(7)^.

### Previous treatment of PDR

In only half of the eyes within this study, PDR had been treated before VH occurred, either by PRP or IVB. This observation is questioning the proper screening and monitoring of DR in patients with DM in our series. The American Academy of Ophthalmology underlines in the Diabetic Retinopathy Preferred Practice Pattern that the risk of severe vision loss or vitrectomy is reduced by 50% in patients treated with PRP^(1)^.The role of PRP is indisputable in controlling the progression of retinopathy by interrupting fibrovascular proliferation and preventing VH^(3)^. It was shown that following PRP, the interleukin-6 vitreous body levels increase causing dysfunction of the endothelial barrier with subsequent macular edema^(8)^. This brings up the issue of whether to perform PRP before surgery to reduce disease activity but with the risk to favor macular edema or during PPV^(8)^. According to our experience, PPV was easier in previously lasered eyes, proportional to the amount of laser treatment. Therefore, even if we intend to perform PPV, we will perform as much retinal laser photocoagulation as possible before PPV. Intravitreal ranibizumab proved to be similar to PRP in terms of efficacy and safety in patients with PDR after 5 years of follow-up with lower rates of vision-impairing DME and less visual field loss^(9)^.

### Antiplatelet drugs

The results of our study suggest that antiplatelet therapy did not increase the risk of rebleeding, as opposed to other studiesr^(10)^. Although delamination for PDR is a risk factor for bleeding following PPV, the severity of thromboembolic events precipitated by the cessation of antiplatelet drugs exceeds the negative visual impact of the VH^(11)^. Other potential complications apart from vitreous cavity hemorrhage are subretinal bleeding and choroidal or suprachoroidal hemorrhage. The authors of the same study recommended reducing the risk of intraocular bleeding by pretreating with IVB rather than stopping antiplatelet drugs^(11)^, which was also in agreement with our attitude.

### Intravitreal bevacizumab

Consistently with the observations of other studies, IVB administered in the preoperative period facilitates surgery, although, with respect to BCVA, it did not influence it significantly in our series. This is in contrast with other reports suggesting that IVB administered before PPV improved BVCA, decreased surgical time^(12)^, and reduced the risk of postoperative bleeding^(13)^. According to our experience, preoperative IVB made surgery easier, allowing the surgeon to perform more precise and efficient motions with less risk for hemorrhage and re­tinal breaks. The risk of early or late postoperative bleeding was not influenced significantly by the moment of IVB administration, i.e., before or at the end of surgery.

When PPV was performed without IVB, a 17%-60% incidence of postoperative VH was reported, as compared to 11%-38% in the group with adjuvant IVB^(14)^. Our results are comparable to those published in the literature, which show the incidence of postoperative bleeding of 13.9% and 23.2% for early- and late-onset VH, respectively.

### Phacoemulsification

Previous reports showed that vitreous gel removal by PPV leads to altered lens permeability and aqueous humor composition and to the intraoperative oxidation of lens proteins, which may accelerate cataract progression in diabetic patients^(15,16)^.

It was observed that if PPV was combined with phacoemulsification, the risk of NVG increased^(17)^, because the lens was thought to represent a protective barrier against anterior segment neovascularization^(16)^.The progression of DR after PPV alone was compared with PPV combined with phacoemulsification, and it was observed that visual acuity was better in the latter group and there was no difference regarding the progression of DR, relapse of VH, and NVG between the two groups^(18)^. According to our results, phacoemulsification combined with PPV was not associated with a higher rate of postoperative NVG. The potential advantages of combined surgery are the avoidance of the second operation for postvitrectomy cataracts, faster recovery of the visual function, and better postoperative visualization of the retina^(19,20)^.

### Preoperative PRP

In our series, 88% of the eyes that had been lasered before PPV needed laser during surgery. This observation raises concerns about the quality of the prior laser treatment in terms of density, number, and/or dimension of laser spots. However, it is often difficult to achieve a complete PRP in cases of chronic VH and fibrovascular proliferation, as in the cases included in this study. Therefore, the preexisting hemorrhage and disease severity may have been the cause of inadequate preoperative laser rather than incorrectness of laser application. If VH develops “shortly following” PRP, within the first 4 weeks, it seems to be due to the contraction of the fibrous component as the fibrovascular membrane regresses and there is no need to add laser treatment^(3)^. On the contrary, if the patient has recurrent episodes of VH, they are most likely due to active neovascularization at the disk or elsewhere^(3)^. Better visual outcomes after PPV in patients previously treated with PRP or IVB have been reported by similar studies^(3)^. The Early Treatment of Diabetic retinopathy study (ETDRS) showed that 5% of eyes belonging to patients with PDR still required vitrectomy despite apparently adequate pan-retinal photocoagulation^(21)^.

### Fibrovascular proliferation

The presence of fibrovascular membranes indicates advanced PDR; therefore, it is obvious that a simpler PPV with no need to peel/dissect fibrovascular tissue from the retinal surface is associated with lower complication rates and better anatomic and functional outcomes.

However, the complexity of the surgery and the subsequent outcome are closely related to the degree and amount of fibrovascular proliferation, whether it is in the macula or adjacent to the optic disc. Even if there is only one fibrovascular proliferation requiring dissection in the equatorial part of the nasal retina, the prognosis of visual acuity is considered good, and many cases have a good prognosis of visual acuity. In our study, the need for dissection of fibrovascular membranes had a significant negative impact on the final BCVA. A previous study highlighted that the surgical outcomes were better in patients with a recent decrease in visual acuity and poorer with longstanding macular heterotopias^(22)^. According to the same study, the visual prognosis seemed to be influenced by several factors, such as patient age, location, and extent of fibrovascular membranes and presence of macular heterotopias^(22)^.

### Intraoperative complications

During dissection, posterior retinal breaks usually occur adjacent to vitreoretinal adhesions, most often where membranes are fibrous and the retina is thin and atrophic^(22)^. In a study of 760 eyes that underwent PPV for PDR, a 28.5% incidence for iatrogenic retinal breaks was found, where posterior breaks had poorer outcomes compared to peripheral breaks or oral dialyzes^(23)^.

We report a relatively low rate of iatrogenic retinal breaks in our study, which was 1.16%. The difference could be explained by more severe cases included in the previously mentioned study, where the subjects were classified into three severity groups: VH only, fibrovascular membranes without TRD, and fibrovascular membranes with TRD. In contrast, our group consisted mainly of eyes with VH and fibrovascular membranes without TRD. In the era of 20G PPV, the risk of iatrogenic retinal breaks was significantly higher, achieving 14%-42%^(14)^. Our hypothesis is that the low rate of intrao­perative retinal breaks was attributed not only to the 23G PPV system itself but also to the use of IVB 3-5 days before surgery in most instances. Therefore, the risk and magnitude of intraoperative bleeding were lower. Intracavity bleeding during PPV obscures the operating field, which could be responsible for higher risk of iatrogenic retinal tears^(24)^. IVB administration within two weeks before PPV seems to be protective against the risk of iatrogenic retinal breaks^(14)^. However, the anti-VEGF crunch syndrome has to be considered. In these cases, IVB induces the regression of the vascular component from the fibrovascular proliferation, but it increases fibrosis, thus, worsening retinal traction. Consequently, once the indication for PPV was set, we injected bevacizumab intravitreally not longer than 5 days before surgery.

### Postoperative complications

Postvitrectomy cataracts developed in 22.09% of the eyes in our study, which is comparable with other data reporting 17%-37% cataracts following PPV for PDR^(22)^. In the present study, NVG was found in 8.72% of the eyes, which is comparable to other reported incidences of 4%-13% for phakic eyes^(22)^. Diabetic patients are relatively immunologically compromised, being predisposed to procedure-related infections, which are also favored by the longer surgery for PDR and multiple insertions and removals of instruments. Hence, we reported one case of endophthalmitis (0.58%). In a 2-year prospective study from the UK, the rate of endophthalmitis after PPV was 0.0058%,^(25)^ which is different from ours, presumably due to the inclusion of other indications for PPV besides diabetic vitreous hemorrhage, such as retinal detachment, macular hole, epiretinal membrane, vitreomacular traction syndrome, vitreous opacity, and nondiabetic VH.

We found a nonstatistically significant difference in the incidence of cataracts with certain tamponade agents, although silicone oil seemed to induce most cataracts in our study. It has been suggested that intraocular tamponade was not superior to no tamponade in reducing postvitrectomy rebleeding, especially in eyes without retinal breaks^(26)^.

### Other risk factors for postoperative rebleeding

Lower extremity amputation and antihypertensive treatment were cited as risk factors for rebleeding in patients with DM that underwent PPV for VH^(27)^. This observation was not confirmed in our study.

### Limitations of the study

Our study has several limitations. Firstly, it is an observational retrospective study. Secondly, we lacked the patients’ glycemic status and their metabolic parameters at the end of follow-up. Thirdly, we also did not follow up with optical coherence tomography of the macula postoperatively. Finally, we only had information about the presence or absence of high blood pressure, which is not a precise value, which could have better indicated the risk of postoperative rebleeding. Moreover, financial constraints prevented us from performing 25 g/27 g PPV.

Nevertheless, this retrospective analysis helped us adjust the management of PDR in controlling associated diseases, stabilizing retinopathy with the help of pan-retinal laser photocoagulation and IVB, and improving the surgical outcome by injecting IVB before or during surgery.

The factors with a significant positive impact on the final BCVA within our study were type 2 DM and the absence of preoperative extensive fibrovascular proliferation. The only factor with a significant negative impact on the final BCVA was postoperative NVG. Previous treatment by PRP or IVB was associated with better final BCVA, but not statistically significant. Final BCVA was not influenced by gender, administration of antiplatelet drugs, tamponade agent, early or late rebleeding, and comorbidities. IVB administration was not significantly associated with better final BCVA and lower rates of rebleeding. Phacoemulsification combined with PPV did not increase the risk of postoperative NVG.
